# Clinical Evolution and Risk Factors in Patients Infected during the First Wave of COVID-19: A Two-Year Longitudinal Study

**DOI:** 10.3390/tropicalmed8070340

**Published:** 2023-06-25

**Authors:** Carlos Rescalvo-Casas, Ramón Pérez-Tanoira, Rocío Fernández Villegas, Marcos Hernando-Gozalo, Laura Seijas-Pereda, Felipe Pérez-García, Helena Moza Moríñigo, Peña Gómez-Herruz, Teresa Arroyo, Rosa González, Cristina Verdú Expósito, Lourdes Lledó García, Juan Romanyk Cabrera, Juan Cuadros-González

**Affiliations:** 1 Departamento de Biomedicina y Biotecnología, Facultad de Medicina, Universidad de Alcalá, 28805 Madrid, Spain; carlosrescalvocasas@gmail.com (C.R.-C.); roziofernandezville@gmail.com (R.F.V.); spflaura@gmail.com (L.S.-P.); felipe.perez.garcia.87@gmail.com (F.P.-G.); cristina.verdu@uah.es (C.V.E.); lourdes.lledo@uah.es (L.L.G.); jromanykc@yahoo.es (J.R.C.); juan.cuadros@uah.es (J.C.-G.); 2 Departamento de Microbiología Clínica, Hospital Universitario Príncipe de Asturias, 28805 Madrid, Spain; marcos.torrejon1998@gmail.com (M.H.-G.); pgherruz@gmail.com (P.G.-H.); tarroyo26@hotmail.com (T.A.); rgonzalezpa@gmail.com (R.G.); 3 Departamento de Química Orgánica y Química Inorgánica, Facultad de Química, Universidad de Alcalá de Henares, 28805 Madrid, Spain; 4 Centro de Investigación Biomédica en Red en Enfermedades Infecciosas (CIBERINFEC), Instituto de Salud Carlos III, 28029 Madrid, Spain; 5 Departamento de Medicina Preventiva y Salud Pública, Hospital Universitario Fundación Jiménez Díaz, 28040 Madrid, Spain; hmorinigo@hotmail.com

**Keywords:** long-COVID-19, risk factors, reinfection, vaccines, coronavirus disease 2019, SARS-CoV-2

## Abstract

A limited number of longitudinal studies have examined the symptoms associated with long-COVID-19. We conducted an assessment of symptom onset, severity and patient recovery, and determined the percentage of patients who experienced reinfection up to 2 years after the initial onset of the disease. Our cohort comprises 377 patients (≥18 years) with laboratory-confirmed COVID-19 in a secondary hospital (Madrid, Spain), throughout March 3–16, 2020. Disease outcomes and clinical data were followed-up until August 12, 2022. We reviewed the evolution of the 253 patients who had survived as of April 2020 (67.1%). Nine died between April 2020 and August 2022. A multivariate regression analysis performed to detect the risk factors associated with long-COVID-19 revealed that the increased likelihood was associated with chronic obstructive lung disease (OR 14.35, 95% CI 1.89–109.09; *p* = 0.010), dyspnea (5.02, 1.02–24.75; *p* = 0.048), higher LDH (3.23, 1.34–7.52; *p* = 0.006), and lower D-dimer levels (0.164, 0.04–0.678; *p* = 0.012). Reinfected patients (*n* = 45) (47.8 years; 39.7–67.2) were younger than non-reinfected patients (64.1 years; 48.6–74.4)) (*p* < 0.001). Patients who received a combination of vaccines exhibited fewer symptoms (44.4%) compared to those who received a single type of vaccine (77.8%) (*p* = 0.048). Long-COVID-19 was detected in 27.05% (66/244) of patients. The early detection of risk factors helps predict the clinical course of patients with COVID-19. Middle-aged adults could be susceptible to reinfection, highlighting the importance of prevention and control measures regardless of vaccination status.

## 1. Introduction

The COVID-19 pandemic had a catastrophic effect on public health in the region of Madrid, Spain. As of 11 March 2022, the disease had caused 27.791 deaths in the region, one of the highest crude mortality rates among hospitalized patients worldwide (19.1%) [1]. Between 3 and 16 March 2020, 377 patients presented to the emergency department of our secondary hospital in Madrid with COVID-19 confirmed by PCR. Their medical records were reviewed to determine the disease outcome until 12 August 2022.

Most individuals infected with SARS-CoV-2 experience mild disease with non-specific symptoms, and a considerable proportion (ranging from 6% to 41%) may remain asymptomatic [2]. However, a subset of patients may deteriorate to a critical condition with a heightened susceptibility to mortality.

While the acute phase of the disease attracted great attention by the scientific community, there has been less interest in the long-term clinical course of the disease and as a result, the knowledge on this topic remains limited [2,3]. The terminology used to refer to long-term clinical sequelae includes terms as “long-COVID-19”, post-COVID-19 manifestations”, and “long-term COVID-19 effects” [3]. To remedy this situation, the World Health Organization (WHO) recently developed a clinical case definition of the so-called post-COVID-19 condition, though other agencies continue to label the syndrome differently. Despite this heterogeneity, the definition of long-COVID-19 varies between 4 and 12 weeks after the acute phase [2].

Previous studies have shown the potential late complications of COVID-19 infection, including psychological, cardiovascular, respiratory, endocrine, hematologic, dermatologic, digestive, renal, and neurological disorders, as well as mood disturbances [4,5]. The effect of this pathology is greater than expected. A study published in the journal *JAMA Network Open* in 2022 examined a cohort of COVID-19 patients and revealed that approximately 30% of them experienced long-term symptoms, even after overcoming the initial infection [6]. In other research, the occurrence rate of long-COVID-19 ranges from 2–13% [7] to 10–81% [8], highlighting a discrepancy in the data. These symptoms include persistent fatigue, respiratory difficulties, sleep disorders, brain fog, and cognitive impairment [6], with a higher prevalence in fatigue, shortness of breath and headache [6,7].

These symptoms have been observed to persist for more than six months after the resolution of illness, impacting the lifestyle and economy of the countries [6]. In contrast, older individuals, those who were hospitalized, and predominantly women exhibited a higher likelihood of experiencing long-COVID-19 [7].

More importantly, efforts are needed to detect risk factors that will enable us to predict a protracted course early in the disease or identify those patients at higher risk of developing long-COVID-19 [5,7].

The wide range of persistent symptoms in long-COVID-19 requires a coordinated response from multiple medical specialties and multidisciplinary collaborations [8]. COVID-19 reinfection has been a topic of interest and concern in the field of health. The natural immunity acquired after initial infection can last for several months, but the efficacy in preventing reinfection may gradually diminish over time [9]. Following infection, the immune response of the body will produce antibodies, memory B cells and T cells [10]. These latter two, in their provision of adaptive immunity [10], shall endure beyond the transient nature of the innate. According to the study conducted by Hil et al., the reinfection rate was observed to be 7.6%, indicating that a significant proportion of individuals are at risk of experiencing a subsequent infection [9]. Furthermore, the reinfection rates per 10,000 individuals were reported as 2.25 for non-vaccinated patients, whereas for vaccinated individuals, the rate was 0.43 [9], within the first year following the primary infection. These findings highlight the importance of considering reinfection as a potential concern, emphasizing the need for continued monitoring and vaccination efforts to mitigate the risk of recurrent infections. This highlights the importance of vaccination as a strategy to strengthen protection against reinfection.

The SARS-CoV-2 virus uses its spike protein to fuse to the cell receptor ACE2 that is present in almost all cells [10]. Cell-mediated immunity against SARS-CoV-2 engenders the development of distinct T-cell subsets that show specificity towards spike proteins and membrane antigens [10]. Therefore, mutations in the SARS-CoV-2 spike protein alter the virus, leading to new disease variants [11]. The Beta variant has resistance against vaccines developed to train the immune system to recognize the spike protein (i.e., AstraZeneca) [12], while mRNA vaccines (i.e., Moderna), which are focused on spike mutations, can increase the number of antibodies produced [11]. Research into the factors associated with an initial infection [11] may provide valuable insight against a second infection.

We aimed to evaluate the clinical course over 29 months of 377 consecutive adult patients (≥18 years) with laboratory-confirmed COVID-19 observed in our hospital during the first outbreak of COVID-19 and to investigate the risk factors associated with increased mortality or greater likelihood of developing long-COVID-19.

## 2. Methods

### 2.1. Study Setting and Design

This study was conducted in a medium-sized hospital that serves approximately 243.000 inhabitants in the region of Madrid, Spain. We retrospectively analyzed cases of COVID-19 in adult patients confirmed in the period spanning 3–16 March 2020. A positive case of COVID-19 was defined as a patient with at least one compatible clinical symptom (fever > −37.5 °C, cough, or dyspnea) and a positive RT-PCR result for SARS-CoV-2 in a nasopharyngeal specimen or lower-respiratory-tract sample (sputum or bronchial aspirate) [13].

A 12-week threshold period was used to study long-COVID-19, as the acute phase included symptoms presented between 4 and 12 weeks, in accordance with the NICE guidelines [1,14].

This study obtained approval from the Ethics Committee of Hospital Universitario Príncipe de Asturias following the acceptance of the manuscript detailing the methodology, timeline, and personnel involved in conducting the study. All authors affirm the precision and comprehensiveness of the data and confirm that the study adheres to the established protocol.

### 2.2. Diagnostic Procedures

SARS-CoV-2 infection was diagnosed according to the WHO protocol [15]. Viral RNA was obtained from clinical samples by 2 automatic extractors, i.e., the MagCore HF16 nucleic acid extractor (RBC Bioscience, Taipei, Taiwan) and the Hamilton Microlab Starlet liquid handler (Hamilton Company, Bonaduz, Switzerland).

Viral RNA was amplified with the use of the following 2 real-time PCR platforms: VIASURE SARS-CoV-2 Real-Time PCR Detection Kit (Certest Biotech, Saragossa, Spain) and Allplex 2019-nCoV assay (Seegene, Seoul, South Korea).

The multiplex (AllplexTM Respiratory Full Panel Assay, Seegene, Seoul, South Korea) RT-PCR detection kit was used to detect other respiratory pathogens.

### 2.3. Inclusion and Exclusion Criteria

For incidence and mortality analysis, all consecutive adult patients with a diagnosis confirmed by RT-PCR in nasopharyngeal, sputum, or lower-respiratory-tract samples in the period spanning 3–16 March 2020 were included; evaluation of the risk factors included all adult patients presenting to the emergency department of our hospital.

Clinical data included age, sex, comorbidities (i.e., hypertension, diabetes mellitus, coronary heart disease, chronic obstructive pulmonary disease (COPD), chronic kidney disease, immunosuppression), symptoms (fever, cough, dyspnea), days from illness onset to hospital admission and laboratory data (lymphocyte count, aspartate aminotransferase (AST), alanine aminotransferase (ALT), lactate dehydrogenase (LDH), D-dimer, ferritin, and interleukin-6 (IL-6), cycle threshold (Ct) values and co-infections detected simultaneously with a COVID-19 diagnosis). Symptoms associated with pre-existing conditions were not considered as COVID-19 symptoms. Only symptoms that manifested concurrently with the COVID-19 infection were taken into consideration for this analysis.

The presence of co-infections was studied in 206 patients for whom samples were available for multiplex respiratory RT-PCR (Allplex TM Respiratory Full Panel Assay).

Treatments included any of the following drugs administered as monotherapy or in combination: antiretroviral treatment (darunavir/cobicistat or ritonavir/lopinavir), chloroquine or hydroxychloroquine, tocilizumab, or type I interferon.

Finally, the medical records of these patients were reviewed until 12 August 2022, to detect reinfection (symptomatic, asymptomatic) and long-COVID-19. Patients who were transferred or for whom only age, sex and positive PCR without any additional information or medical history were available were excluded from the study, as it was determined that crucial variables were missing from the analysis. Certain data from those patients could not be retrieved due to the absence of the pertinent test or the unavailability of test results in their medical records. Furthermore, among the 377 patients, 124 of them died, leading to the impossibility of acquiring their data for analysis on reinfection and long-COVID-19. The study may be subject to biases. The population selection process excluded all underage patients and those for whom we lacked the necessary information for the study. This may have resulted in a non-representative population of the specific region under investigation.

### 2.4. Study Definitions and Outcome Description

The WHO criteria were used to classify the severity of infection as mild disease (uncomplicated disease, including non-specific symptoms); pneumonia (no signs of severe pneumonia and no need for supplemental oxygen); severe pneumonia (fever or suspected respiratory infection plus one of the following: respiratory rate > 30 breaths/min; severe respiratory distress or SpO_2_ ≤ 93% on room air) [16].

Immunosuppression at the time of presentation was determined in any of the following conditions: administration of corticosteroids or another immunosuppressive drug within the previous month, or ongoing antineoplastic treatment. Patient discharge criteria were normal body temperature for 3 days, 2 negative PCR results at a 24 h interval, and resolution of all clinical symptoms.

### 2.5. Statistical Analysis

Continuous variables appear as the median and interquartile range (IQR) and categorical variables as frequencies. We used the Mann–Whitney U-test, χ^2^ test, or Fisher’s exact test to compare differences between survivors and non-survivors where appropriate. A *p* value of 0.05 or below was considered significant. Statistical analysis was performed with SPSS v20.0 (IBM Corp., Armonk, NY, USA).

Univariate and multivariate logistic regression models were used to analyze the risk factors associated with in-hospital death and patient evolution. The logistic regression model was adjusted for the most significant variables, with results expressed as odds ratios (OR) and 95% confidence intervals (CI). Considering the 377 patients, 9 variables were chosen for multivariate analysis based on previous findings and clinical constraints.

## 3. Results

A total of 494 adult patients were diagnosed with SARS-CoV-2 infection in our hospital in the study period (3–16 March 2020), 124 of whom died, which results in a crude mortality rate of 25.1% (124/494). Considering the hospital catchment area of 249.578 inhabitants, we determined an accumulative disease incidence and mortality rate of 225/100 000; 43/100.000, respectively.

For the risk factor analysis, all 377 patients who visited the emergency department were included; 117 additional patients examined in the hospital department of occupational health or in primary care facilities were excluded due to a lack of key information in their medical records. Table 1 summarizes the demographic and clinical findings of the patients.

Advanced age, specifically individuals aged 65 and above, was identified as a risk factor for mortality among COVID-19 patients (Table 1). Furthermore, elevated viral load (measured by Ct value), the presence of comorbidities (excluding chronic kidney disease), symptoms such as pneumonia and dyspnea, and abnormal laboratory results (including lymphocyte count, ALT, LDH, and D-dimer) were all associated with a higher probability of death among the patients (Table 1). Co-infection was detected in 60 (29.1%) of the 206 for multiplex respiratory RT-PCR (Table 1). We searched for respiratory and non-respiratory co-infections at diagnosis and hospital-acquired infection, but found no further significant differences between the survivors and non-survivors (*p* = 0.950 and 0.877 respectively). The most common pathogens responsible for co-infection upon hospital admission were *Haemophilus influenzae* (respiratory sample; *n* = 17), *Escherichia coli* (urine sample; *n* = 8), and coagulase-negative *Staphylococcus* (blood culture; *n* = 5).

In April 2020, 180 (47.7%) patients had received antibiotics, 178 (47.2%) antiretroviral drugs and 160 (42.4%) chloroquine/hydroxychloroquine, tocilizumab, or type I interferon. Non-survivors had received more antibiotic treatments (78 (62.9%)) compared with survivors (102 (40.3%)) (*p* < 0.001).

A univariate analysis of disease outcome as of April 2020 revealed that the odds of in-hospital death were higher in patients with comorbidities, higher age, viral load (lower Ct values), LDH, ferritin, and lymphopenia (Table 2).

Multivariate regression showed an increased probability of in-hospital death associated with age over 65 (OR 5.48, 95% CI 2.21–13.58; *p* < 0.001), lower Ct values (0.89, 0.84–0.96; *p* ≤ 0.001), coronary heart disease (2.66, 1.30–5.44; *p* < 0.007), and both lower lymphocyte count (0.34, 0.17–0.68; *p* = 0.002) and higher LDH (1.44, 1.17–1.77; *p* ≤ 0.001) per 1-unit decrease and 100-unit increase, respectively (Table 2).

The median time until reinfection was 770 days (IQR 661–820) after first diagnosis; reinfected patients (*n* = 45) (47.8 years; 39.7–67.2) were younger than non-reinfected patients (64.1 years; 48.6–74.4)] (*p* < 0.001). Reinfection was highest among patients between the ages of 40 and 49 years (29.7%).

Of the 45 reinfected patients, 4 were unvaccinated, 27 were fully vaccinated, and 14 had a partial vaccination schedule (Table 3). All reinfections were diagnosed from December 2021 to August 2022. In unvaccinated patients, the time to reinfection was shorter and most of them had symptoms without statistical significance. (Table 3). Less patients who received combined vaccines developed COVID-19-related symptoms (44.4%) compared to those vaccinated with only one type (77.8%; *p* = 0.048). No statistically significant differences were found for demographic and epidemiological characteristics between the vaccinated and unvaccinated patients.

We reviewed the evolution of the 253 patients who had survived from April 2020 to 12 August 2022 (Table 4). Each column represents the number of patients exhibiting that specific characteristic. Nine died between April 2020 and August 2022. A total of 45 (17.8%) patients were reinfected with SARS-CoV-2, with 4 being asymptomatic. The most common symptoms were fever and cough (61.0% for both) (Table 4a). Only seven of the patients who suffered reinfection were treated in the hospital emergency department for suspected COVID-19 and were hospitalized. Long-COVID-19 was detected in 66 (26.1%) patients, with dyspnea as the most common symptom (62.1%), followed by fatigue (18.2%) (Table 4b). After April 2020, a total of 147 patients returned to the emergency department; 43 (29.3%) were patients with long-COVID-19, 28 of these presenting symptoms common to COVID-19. Among the 19 hospitalized patients who experienced long-COVID-19, only one was specifically admitted due to long-COVID-19 syndrome.

The demographic and clinical findings on long-COVID-19 patients are shown in Table 5a. The risk factors associated with developing long-COVID-19 were reduced Ct levels in the SARS-CoV-2 diagnostic PCR, cardiovascular disease, chronic lung disease, pneumonia, dyspnea, disease severity, longer time from disease onset to hospital admission and lower D-dimer levels. A univariate analysis performed to detect risk factors associated with long-COVID-19 (Table 5b) showed increasing odds of developing the syndrome for patients in emergency care with cardiovascular disease, chronic obstructive lung disease, pneumonia, dyspnea, higher LDH, and lower D-dimer levels. A multivariate regression analysis revealed that the increased likelihood was associated with chronic obstructive lung disease (OR 14.35, 95% CI 1.89–109.09; *p* = 0.010), dyspnea (5.02, 1.02–24.75; *p* = 0.048), higher LDH (3.23, 1.34–7.52; *p* = 0.006), and lower D-dimer levels (0.164, 0.04–0.678; *p* = 0.012).

## 4. Discussion

Knowledge of the long-term clinical course of individuals infected with COVID-19 remains limited. The main findings of this study concern risk factors for long-COVID-19, the reinfection rate among younger people, factors associated with reinfection such as vaccination status and existing risk factors for mortality at the time of infection.

Although the long-term complications of COVID-19 vary widely, we found that certain patient attributes could predict long-COVID-19, such as cardiovascular disease, chronic obstructive lung disease, pneumonia, dyspnea, higher LDH, and lower D-dimer levels. Our findings are consistent with those of other researchers, including Hill, E et al., who concluded that chronic lung disease was associated with an elevated risk of long-COVID-19 [4,17], while other risk factors reported by the authors, such as obesity or race, were not measured in our cohort [17].

The long-COVID-19 syndrome may be associated with 26 of the 29 proteins encoded by the SARS-CoV-2 genome that induce changes in endothelial permeability, affecting tight junction proteins such as cadherin 1–5 [18]. However, additional factors may also play a role, which would explain the disparate symptoms found here.

The clinical presentation of COVID-19 has been shown to vary [4,19]. Our data support this, as 39.4% of the patients studied developed symptoms such as hair loss, neuromuscular problems, blurry vision, dizziness, and cognitive impairment. Nonetheless, respiratory complications remain the hallmark of COVID-19 (53.0%). These results are consistent with previous reports on long-COVID-19 [20]. The rate of long-COVID-19 in our study was 27.1%, as compared to 2.3–37% in some studies [21] and 30–50% in others [19].

In our cohort, a higher proportion of patients were reinfected with SARS-CoV-2 (17.9%) than in other studies [12,22]. Diagnosis occurred from December 2021 to August 2022, during the Omicron variant wave [12,23], which indicates its capacity to neutralize vaccine activity [24].

Forty-one reinfected patients had received at least one vaccine dose, where only six of whom required hospitalization related to COVID-19. Combining COVID-19 vaccines could reduce symptoms and disease severity [25]. Even though N. Bechman et al. [26] found that some variables such as sex could play a role in the efficiency of the vaccines against COVID-19, this sex dimorphism was not observed in our study. On the other hand, studies investigating patients receiving mixed vaccine regimens are limited [25,27], which further emphasizes the significance of the reduced symptomatology in patients who received a combination of vaccines [25,27]. Our results show a statistically significant relationship between age and reinfection, as subsequent infection was more common in middle-aged adults (median age of 47.8 years) than older patients (median age of 64.1 years) with similar tendencies in other studies [23].

The mortality rate in patients presenting to the emergency department was higher than studies conducted in China (6–11%) [15,28]. This difference may result from the difference in the cohort comorbidities (65.0%) compared to a 32% in the cohort of Huang et al. [28].

The main risk factors for patients with SARS-CoV-2 infection were age over 65 years and comorbidities, especially hypertension, followed by heart disease [16], diabetes [13], higher LDH levels, lymphopenia, lower D-dimer levels, higher viral load [29,30], COPD, pneumonia, higher ferritin, and dyspnea. Contrasting with other reports [13,31,32], we found no relation between death and co-infection [31,33], male sex [13], higher D-dimer, or dyspnea [32]. Multivariate logistic regression analysis revealed advanced age to be a strong predictor of death from COVID-19, which supports the findings of Du et al. [34]. In our cohort, SARS-CoV-2 induced community-acquired pneumonia characterized by lymphopenia, thus supporting earlier findings [35] and suggesting a role played by immune dysregulation in this critical illness.

Whether these factors are replicated in other populations needs to be assessed in further studies. If similar results are obtained, these factors could be used as predictors to individualize patient management. Early recognition of these and other risk factors could help identify patients at risk of severe disease.

Our research has some limitations. Firstly, due to the retrospective study design, not all laboratory tests were performed in all patients. Secondly, some patients were transferred to other facilities, meaning we were unable to study their outcomes. Third, reinfections in patients were only investigated in those who returned to a healthcare center and presented symptoms compatible with COVID-19. In addition, we may have underestimated the number of cases due to inconsistencies in the frequency of collection of respiratory samples and a relatively low positive rate of detection from RNA in throat swabs. Another limitation of this study lies in the difficulty in deciding whether the COVID-19 compatible symptoms for which a patient comes to the emergency department for are due to their comorbidities. Lastly, because of all the limitations in our research, we are unable to conclude that the results given are appliable for all populations. Therefore, further studies are necessary to determine the applicability of the findings. In future studies, greater consideration will be given to the number of individuals within each distinct age group, ethnicity, and gender to achieve a more representative sample of the overall population.

## 5. Conclusions

This study shows that the COVID-19 epidemic had a major impact in our community, with a high cumulative incidence and mortality rate in the period studied. Advanced age, Ct values, cardiovascular disease, a lower lymphocyte count, and higher levels of LDH were associated with increased mortality. The combination of all the above factors increases the likelihood of developing long-COVID-19 or death.

Research into the evolution of COVID-19 cases may aid in the prognosis and treatment of this emerging disease, which currently affects more than a quarter of patients. The rate of SARS-CoV-2 reinfection among younger people was higher than that of initial infection, and further research should confirm this finding and its causes.

Administration of combined vaccines produced a decrease in symptoms for reinfected COVID-19 patients. Nonetheless, we believe there is a clear need for variant-adapted vaccines and to maintain public health measures to control the spread of emerging variants.

## Figures and Tables

**Table 1:** Demographic and clinical characteristics of patients on admission.

	Total (%)	Non-Survivors (%)	Survivors (%)	*p* Value
**Patients**	377	124 (32.9)	253 (67.2)	
**Median age in years (IQR)**	71.52 (50.76–80.74)	81.6 (74.55–86.93)	60.13 (47.19–73.91)	**<0.001**
**Age ≥ 65 years (%)**	231 (61.3)	120 (90.2)	111 (45.5)	**<0.001**
**Male sex (%)**	195 (51.7)	69 (55.6)	126 (49.8)	0.286
**Ct (*n* = 336)**	26.4 (22.4–30.1)	24.84 (21.01–28.06)	27.25 (23.3–30.8)	**<0.001**
**Comorbidities (%)**	245 (65.0)	111 (89.5)	134 (53.0)	**0.001**
** Hypertension (%)**	168 (44.6)	80 (64.5)	88 (34.8)	**<0.001**
** Diabetes (%)**	93 (24.7)	42 (33.9)	51 (20.2)	**0.004**
** Cardiovascular disease (%)**	98 (26.0)	61 (49.2)	37 (14.6)	**<0.001**
** COPD (%)**	59 (15.6)	31 (21.0)	28 (11.1)	**<0.001**
** Carcinoma (%)**	51 (13.5)	28 (22.6)	23 (9.1)	**<0.001**
** Chronic kidney disease (%)**	36 (9.5)	17 (13.7)	19 (7.5)	0.054
** Immunosuppression (%)**	30 (8.0)	17 (13.7)	13 (5.1)	**0.004**
**Fever (temperature ≥ 37.5 °C) (%)**	256 (67.9)	82 (66.1)	174 (68.8)	0.605
**Co-infection (*n* = 206)**	60 (29.1)	20 (16.1)	40 (15.8)	0.95
**Cough (%)**	230 (61.0)	77 (62.1)	153 (60.5)	0.762
**Pneumonia (%)**	270 (71.6)	109 (87.9)	161 (63.6)	**<0.001**
**Dyspnea (%)**	199 (52.8)	89 (71.8)	110 (43.5)	**<0.001**
**Disease severity**				<0.001 *
** Mild disease or non-severe pneumonia (%)**	103 (27.3)	10 (8.1)	93 (36.8)	
** Severe pneumonia (%)**	137 (36.3)	24 (19.4)	113 (44.7)	
** Critical (%)**	137 (36.3)	90 (72.6)	47 (18.6)	
**Time from disease onset to hospital admission, days**	3.0 (1–6)	3 (1–5)	3 (1–7)	0.06
**Laboratory findings**				
**Lymphocyte count × 10^3^/µL (*n* = 371)**	0.82 (0.58–1.21)	0.69 (0.47–1.02)	0.9 (0.66–1.29)	**<0.001**
**AST, U/L (*n* = 214)**	32.0 (22.0–45.0)	32.0 (21.0–52)	32.0 (22.0–45.0)	0.771
**ALT, U/L (*n* = 287)**	25.0 (18.0–41.75)	23.0 (14.5–36.0)	26.0 (19.0–43.0)	**0.021**
**LDH, U/L (*n* = 300)**	295.0 (225.5–378.0)	318.0 (248.0–435.25)	284.0 (211.0–339.0)	**≤0.001**
**Ferritin (ng/mL) (*n* = 72)**	539.0 (233.0–955.0)	656.0 (339.0–1780.5)	462 (217.75–829.5)	0.065
**D-dimer (µg/L) (*n* = 98)**	1.05 (0.47–1.85)	1.52 (1.01–2.2)	0.76 (0.4–1.71)	**0.002**

Statistics: Values are expressed as median (IQR) and absolute count (percentage). *p*-values calculated by Mann–Whitney U test, χ2 test, or Fisher’s exact test, as appropriate. Significant differences in bold. * χ2 test comparing all subcategories. Abbreviations: IQR, interquartile range; AST, aspartate aminotransferase; ALT, alanine aminotransferase; COPD, chronic obstructive pulmonary disease; LDH, lactate dehydrogenase; U/L, units per liter; ng/mL, nanograms per milliliter; µg/L, micrograms per liter.

**Table 2:** Risk factors associated with in-hospital death.

	Univariate OR (95% CI)	*p* Value	Multivariate OR	*p* Value
			(95% CI)	
**Demographic and clinical characteristics**				
Age, years *	1.11 (1.09–1.11)	**<0.001**		
**Age > 65 years**	11.75 (6.04–22.89)	**<0.001**	5.48 (2.21–13.58)	**<0.001**
**Sex, male vs. female**	0.79 (0.51–1.22)	0.287		
**Ct (*n* = 336)**	0.91 (0.87–0.96)	**<0.001**	0.89 (0.84–0.96)	**≤0.001**
**Comorbidity presence (vs. non-presence)**				
** Hypertension**	3.41 (2.17–5.35)	**<0.001**	1.13 (0.56–2.26)	0.734
** Diabetes**	2.03 (1.25–3.23)	**0.004**	1.13 (0.54–2.34)	0.745
** Cardiovascular disease**	5.65 (3.44–9.28)	**<0.001**	2.66 (1.30–5.44)	**0.007**
** Chronic obstructive pulmonary disease**	2.68 (1.52–4.71)	**0.001**	1.28 (0.57–2.89)	0.558
** Carcinoma**	2.92 (1.60–5.32)	**<0.001**	2.30 (0.96–5.50)	0.062
** Chronic kidney disease**	1.96 (0.98–9.13)	0.058		
** Immunosuppression**	2.93 (1.38–6.25)	**0.005**	1.66 (0.54–5.11)	0.374
** Fever (temperature** **≥ 37.3 °C)**	0.89 (0.56–1.40)	0.605		
** Cough**	1.07 (0.69–1.66)	0.762		
** Pneumonia**	4.15 (2.29–7.55)	**<0.001**		
** Dyspnea**	3.31 (2.08–5.25)	**<0.001**		
**Laboratory findings**				
Lymphocyte count × 10^3^/µL (*n* = 3) *	0.54 (0.35–0.83)	**0.005**	0.34 (0.17–0.68)	**0.002**
AST, U/L (*n* = 214) *	1.00 (0.99–1.01)	0.828		
**ALT, U/L (*n* = 287)**	0.99 (0.99–1.01)	0.845		
LDH, U/L (*n* = 300) **	1.29 (1.11–1.50)	**≤0.001**	1.44 (1.17–1.77)	**≤0.001**
Ferritin (ng/mL) (*n* = 73) *	1.00 (1.00–1.00)	**0.041**		
D-dimer (µg/L) (*n* = 98) *	1.01 (0.86–1.20)	0.887		
**Co-infection diagnosis COVID-19**	1.02 (0.54–1.93)	0.95		

Univariate regression was performed for all the demographic and clinical characteristics. Multivariate regression for: age > 65 years, Ct, hypertension, diabetes, cardiovascular disease, chronic obstructive pulmonary disease, carcinoma, immunosuppression, lymphocyte count, LDH. OR = odds ratio. ALT = alanine aminotransferase. AST = aspartate transaminase. U/L = units per liter. * Per 1-unit decrease. ** Per 100-unit increase.

**Table 3:** Vaccination status of reinfected patients.

	COVID-19 Symptoms	*p*-Value	Time in Days to Reinfection	Time from Last Vaccine Dose to Reinfection (Days)
**Unvaccinated (*n* = 4)**	3 (75.0)	*p* = 0.857	719.5 (684.5–807)	-
**Vaccinated (*n =* 41)**	29 (70.7)		772.0 (664.5–820)	166.0 (85–216)
** ** **Non-combined**	28 (77.8)	*p* = 0.048	767.0 (666–820.5)	185.5 (98.5–223)
** ** **Combined**	4 (44.4)		775.0 (709.5–809.5)	148.0 (54–167.5)
Pfizer-AstraZeneca	1 (25.0)		770.0 (765–770)	114.0 (74–154)
Pfizer-Moderna	2 (50.0)		771.0 (657.75–801.75)	122.0 (32.75–166.75)
AstraZeneca-Moderna	1 (25.0)		835.0 (675.75-701.75)	204.0 (204.0)

**Table 4:** Evolution of COVID-19 survivors from April 2020 to 12 August 2022.

(**a**) Evolution of reinfected COVID-19 survivors									
			**Total (%)**	**Hospitalization** **(*n* = 53)**	**ICU Admission** **(*n* = 2)**	**Emergency Department Visit** **(*n* = 147)**	**COVID-19-Related Emergency (*n* = 8)**	**Non-COVID-19-Related Emergency (*n* = 100)**	**Emergency with COVID-19-like Symptoms but Negative COVID-19 Test (*n* = 39)**
**Reinfection (*n* = 244)**			45 (17.86)	7 (13.2)	0	27 (19.3)	7 (87.5)	16 (16.0)	4 (10.3)
	**Non-symptomatic**		4 (8.9)	1 (1.9)	0	3 (11.1)	1 (14.3)	1 (6.2)	1 (25.0)
	**Symptomatic**		41 (91.1)	6 (11.3)	0	24 (88.9)	6 (85.7)	15 (93.8)	3 (75.0)
		**Fever**	25 (61.0)	3 (50.0)	0	15 (62.5)	4 (66.7)	8 (53.3)	3 (100.0)
		**Cough**	25 (61.0)	2 (33.3)	0	15 (62.5)	3 (50.0)	9 (0.6)	3 (100.0)
		**Pneumonia**	1 (2.4)	0	0	1 (4.2)	1 (16.7)	0	0
		**Dyspnea**	0	0	0	0	0	0	0
		**Asthenia**	4 (9.8)	1 (16.7)	0	2 (8.3)	1 (16.7)	1 (6.7)	0
(**b**) Evolution of long-COVID-19 survivors									
**Long-COVID-19 syndrome**			66 * (27.05)	19 (35.8)	1 (50.0)	43 (29.3)	1 (12.5)	14 (14.0)	28 (71.8)
**(*n* = 244)**									
	**Pneumonia**		4 (6.1)	2 (10.5)	0	4 (9.3)	0	2 (14.3)	2 (7.1)
	**Cough**		5 (7.6)	1 (5.3)	0	3 (7.0)	0	0	3 (10.7)
	**Fever**		2 (3.0)	0	0	2 (4.7)	0	0	2 (7.1)
	**Dyspnea**		41 (62.1)	13 (68.4)	0	27 (62.8)	1 (100.0)	8 (57.1)	18 (64.3)
	**Asthenia**		10 (15.2)	4 (21.1)	1 (100.0)	6 (13.9)	0	1 (7.1)	5 (17.9)
	**Dysgeusia**		4 (6.1)	0	0	3 (7.0)	0	0	3 (10.7)
	**Parosmia**		4 (6.1)	0	0	3 (7.0)	0	0	3 (10.7)
	**Fatigue**		12 (18.2)	4 (21.1)	0	9 (20.9)	1 (100.0)	2 (14.3)	6 (21.4)
	**Myalgia**		3 (4.5)	1 (5.3)	0	3 (7.0)	1 (100.0)	1 (7.1)	1 (3.6)
	**Headache**		7 (10.6)	1 (5.3)	0	4 (9.3)	1 (100.0)	1 (7.1)	2 (7.1)
	Other **		26 (39.4)	5 (26.3)	1 (100.0)	15 (34.9)	1 (100.0)	5 (35.7)	9 (32.4)

** Other symptoms include hair loss, neuromuscular problems, blurry vision, dizziness, and cognitive impairment. * Patients with long-COVID-19 had more than one symptom.

**Table 5:** (**a**) Demographic characteristics and clinical outcomes of patients with long-COVID-19; (**b**) risk factors associated with long-COVID-19.

(a)						
		Patients without Long-COVID-19 (*n* = 187)		Patients with Long-COVID-19 (*n* = 66)		*p* Value
**No. of patients**		187 (73.9)		66 (26.1)		-
**Median age in years (IQR)**		58.7 (45.2–75.4)		65.3 (50.6–74.0)		-
**Age ≥ 65 years (%) (*n* = 118)**		84 (44.9)		66		0.356
**Sex (male) (%)**		93 (49.7)		33 (50)		0.97
**Ct (cycle threshold)**		27.9 (23.1–30.2)		29.1 (23.9–32.4)		**0.05**
**Comorbidities (%)**		94 (50.3)		40 (60.6)		0.148
** Hypertension (%)**		61 (32.6)		27 (40.9)		0.224
** Diabetes (%)**		41 (21.9)		10 (15.2)		**0.238**
** Cardiovascular disease (%)**		22 (11.8)		15 (22.7)		**0.03**
** Chronic obstructive pulmonary disease**		13 (7.0)		15 (22.7)		**<0.001**
** Carcinoma (%)**		18 (9.6)		5 (7.6)		0.618
** Chronic kidney disease (%)**		12 (6.4)		7 (10.6)		0.267
** Immunosuppression (%)**		8 (4.3)		5 (7.6)		0.297
**Fever on admission (temperature ≥ 37.5 °C) (%)**		123 (65.8)		51 (77.3)		0.083
**Co-infection**		39 (20.9)		14(21.2)		0.586
**Cough on admission (%)**		108 (57.8)		45 (68.2)		0.136
**Pneumonia on admission (%)**		80 (42.7)		54 (81.8)		**<0.001**
**Dyspnea on admission (%)**		69 (36.9)		41 (62.1)		**<0.001**
**Disease severity status**		-		-		**<0.001**
** Mild disease or non-severe pneumonia (%)**		80 (42.8)		13 (19.7)		
** Severe pneumonia (%)**		84 (44.9)		29 (43.9)		
** Critical (%)**		24 (12.8)		24 (36.4)		
**Time from disease onset to hospital admission, days**		3 (1–6)		5 (3–7)		**0.036**
**Laboratory findings**						
**Lymphocyte count × 10 ^3^, /µL (*n* = 65)**		0.9 (0.61–1.3)		0.9 (0.7–1.27)		0.77
**AST, U/L** (*n* = 45) *		32 (22.3–45)		32.0 (21.5–44.5)		0.967
**ALT, U/L** **(*n* = 55)**		25.5 (18.0–42.8)		27.0(20.0–48.0)		0.569
**LDH, U/L** (*n* = 61) **		133 (2.72 (3.31–2.04)		3.03(2.2–4.02)		0.128
Ferritin (ng/mL) (*n* = 33) *		496(216.5–823.0)		394.0(224.0–876.5)		0.932
D-dimer (µg/L) (*n* = 43) *		43 (0.94 (0.94–2.63)		23 (0.47(0.34–1.02)		**0.022**
**Statistics**: Values are expressed as median (IQR) and absolute count (percentage). *p*-values calculated by Mann–Whitney U test, χ^2^ test, or Fisher’s exact test, as appropriate. Significant differences in bold. * χ^2^ test comparing all subcategories. **Abbreviations**: IQR, interquartile range; AST, aspartate aminotransferase; ALT, alanine aminotransferase; LDH, lactate dehydrogenase.						
**(b)**						
	**Univariate OR (95% CI)**		***p* Value**		**Multivariate OR**	***p* value**
					**(95% CI)**	
**Demographic and clinical characteristics**						
Age, years *	1.013 (0.996–1.031)		0.139			
**Age > 65 years**	1.303 (0.743–2.286)		0.356			
**Sex, male vs. female**	0.989 (0.564–1.734)		0.97			
**Ct (*n* = 336)**	1.062 (0.998–1.130)		0.058			
**Comorbidity presence (vs. non-presence)**						
** ** **Hypertension**	1.430 (0.802–2.549)		0.225			
** ** **Diabetes**	0.636 (0.298–1.355)		0.241			
** ** **Cardiovascular disease**	2.206 (1.066–4.566)		**0.033**		0.902 (0.138–5.894)	0.915
** ** **Chronic obstructive lung disease**	3.937 (1.759–8.810)		**<0.001**		14.347 (1.887–109.088)	**0.01**
** ** **Carcinoma**	0.770 (0.274–2.162)		0.619			
** ** **Chronic kidney disease**	1.730 (0.651–4.600)		0.272			
** ** **Immunosuppression**	1.834 (0.578–5.818)		0.303			
** Fever (temperature** **≥ 37.3 °C)**	1.769 (0.923–3.389)		0.085			
** ** **Cough**	1.567 (0.866–2.838)		0.138			
** ** **Pneumonia**	3.364 (1.689–6.703)		**<0.001**		0.263 (0.034–2.037)	0.201
** ** **Dyspnea**	2.805 (1.571–5.006)		**<0.001**		5.017 (1.017–24.753)	**0.048**
**Laboratory findings**						
Lymphocyte count × 10^3^/µL (*n* = 371) *	1.225 (0.774–1.938)		0.386			
AST, U/L (*n* = 214) *	1.005 (0.992–1.017)		0.48			
**ALT, U/L (*n* = 287)**	1.002 (0.992–1.011)		0.73			
LDH, U/L (*n* = 300) **	1.294 (1.024–1.635)		**0.031**		3.232 (1.389–7.519)	**0.006**
Ferritin (ng/mL) (*n* = 73) *	1.000 (0.998–1.001)		0.515			
D-dimer (µg/L) (*n* = 98) *	0.405 (0.182–0.901)		**0.027**		0.164 (0.040–0.678)	**0.012**
**Co-infection diagnosis**	1.391(0.597–3.239)		0.444			

OR = odds ratio. ALT = alanine aminotransferase. AST = aspartate transaminase. * Per 1-unit increase. ** per 100-unit increase.

## Data Availability

Data cannot be shared publicly because they are confidential. Data are available from the Departamento de Microbiología Clínica, Hospital Universitario Príncipe de Asturias for researchers who meet the criteria to access confidential data.
